# The Utility and Limitations of Universal Polymerase Chain Reaction Screening for SARS-CoV-2 During Hospital Admission

**DOI:** 10.7759/cureus.61470

**Published:** 2024-05-31

**Authors:** Naruhiko Ogo, Satoshi Ikegame, Taeko Hotta, Keiko Kan-o, Yasuto Yoneshima, Yoshimasa Shiraishi, Kazuya Tsubouchi, Kentaro Tanaka, Isamu Okamoto

**Affiliations:** 1 Department of Respiratory Medicine, Graduate School of Medical Sciences, Kyushu University, Fukuoka, JPN; 2 Department of Clinical Chemistry and Laboratory Medicine, Kyushu University Hospital, Fukuoka, JPN

**Keywords:** false-positive, pcr screening, covid-19, coronavirus disease, sars-cov-2

## Abstract

Objective: Universal polymerase chain reaction (PCR) screening for severe acute respiratory syndrome coronavirus 2 (SARS-CoV-2) on hospital admission is an effective approach to preventing coronavirus disease 2019 (COVID-19) outbreaks in medical facilities. However, false-positive test results due to a recent infection are a concern. We investigated the usefulness and limitations of universal PCR screening for SARS-CoV-2 on hospital admission in a real-world setting.

Methods: We retrospectively analyzed 1320 attempted hospital admissions for 775 patients at the Department of Respiratory Medicine, Kyushu University Hospital, between January 1, 2022, and May 2, 2023.

Results: Thirty-nine out of 1201 PCR tests (3.2%) yielded a positive result, with 22 of these results being considered false positives on the basis of a recent infection. We found that 39% of cases showed a positive PCR result between 31 and 60 days after the onset of COVID-19, although the threshold cycle (Ct) for target 1 (ORF1ab gene) of the Cobas SARS-CoV-2 test (Roche Diagnostics, Basel, Switzerland) was >30 in most instances.

Conclusion: Hospital admission based on the results of PCR screening for SARS-CoV-2 should take into account not only PCR positivity but also the Ct value and recent COVID-19 history.

## Introduction

Severe acute respiratory syndrome coronavirus 2 (SARS-CoV-2) emerged as a novel zoonotic viral pathogen in 2019 [1] and spread rapidly worldwide as a result of its high infectivity [2]. Effective vaccination subsequently reduced the number of symptomatic infectious events and their severity [3], although coronavirus disease 2019 (COVID-19) can still be fatal among high-risk groups such as the elderly, the immunocompromised, and individuals with chronic respiratory disease [4]. This susceptibility of patients with comorbidities makes it important to minimize the spread of SARS-CoV-2 infection in the hospital setting.

Measures to prevent SARS-CoV-2 infection in hospitals include patient and staff screening, hand hygiene, isolation of patients with a suspected infection, and vaccination [5]. Among these measures, screening of patients on admission by polymerase chain reaction (PCR) [6,7] or antigen [8,9] tests has been found to be particularly effective. It can be difficult to diagnose COVID-19 on the basis of symptoms alone among individuals with other respiratory diseases, given the similarity in symptoms between these conditions. Our Department of Respiratory Medicine mostly treats patients with pulmonary diseases such as lung cancer, interstitial pneumonia, and nontuberculous mycobacterial infections. PCR is quite sensitive and specific for the detection of SARS-CoV-2 [10]. We adopted a policy to perform PCR screening for hospital admission at the beginning of 2022 in order to identify and isolate patients infected with SARS-CoV-2. However, we have experienced false-positive cases, presumably due to previous infections. We have retrospectively analyzed the effectiveness and limitations of our universal PCR screening approach.

## Materials and methods

We retrospectively included 775 hospitalized patients in the Department of Respiratory Medicine at Kyushu University Hospital from January 1, 2022, to May 2, 2023. Inclusion criteria include individuals ≥20 years old who entered or attempted to enter the Department of Respiratory Medicine at Kyushu University Hospital and received PCR tests from January 1, 2022, to May 2, 2023. The exclusion criterion is the refusal to participate in our study of the subjects. According to the implemented quarantine policy, we performed SARS-CoV-2 screening mostly with a nucleic acid amplification test (NAAT) 1 day before scheduled hospital admission during the study period. Several NAATs were available in our hospital, including the Cobas SARS-CoV-2 Test (Roche Diagnostics) performed on the Cobas 6800/5800 system (Cobas PCR), ID NOW COVID-19 Assay (Abbott Laboratories, North Chicago, IL), and Xpert Xpress SARS-CoV-2 Cepheid (Beckman Coulter, Brea, CA). The ID NOW and Xpert Xpress assays were not considered screening tests in this study. Specimens were collected with a nasopharyngeal swab and then subjected to the NAAT. We analyzed the total of 1320 hospital admission attempts for the 775 patients, which included failed attempts because of a positive PCR result. In addition to the screening for hospital admission, we also collected all other Cobas PCR results for the 775 hospitalized patients who were the same as above during the study period in order to track the changes in the cycle threshold (Ct) value of Cobas PCR. A Ct value of less than 40 in target 1 of the Cobas PCR test is defined as a positive PCR result. If the PCR test is unable to detect the target within 40 cycles, it is considered a negative PCR result. PCR-positive cases upon hospital admission fall into three categories as defined in this study: COVID-19, false-positive, and indeterminate cases. We reviewed medical records to make a final determination. When PCR-positive cases exhibited symptoms suggestive of COVID-19 (such as throat pain, cough, and fever), they were classified as COVID-19 cases. For PCR-positive cases without current symptoms suggestive of COVID-19 (such as throat pain, cough, and fever), we questioned the previous history of COVID-19 and recent symptoms suggestive of COVID-19. Cases without a recent COVID-19 diagnosis in the last four months and symptoms were defined as 'PCR-positive cases indeterminate for COVID-19'. In cases without a COVID-19 diagnosis and recent symptoms, we defined these cases as 'PCR-positive cases indeterminate for COVID-19'. We used GraphPad Prism version 10.1.2 (GraphPad Software, Inc., La Jolla, CA) to draw a simple linear regression (least squares method), plot the 95% confidence interval, and calculate the r-value of the regression line.

This study was approved by the ‘Institutional Review Boards/Ethics Committees of Kyushu University Hospital and Medical Institutions’ on November 1, 2023 (approval number 23253-00). Patient consent was waived in accordance with the ethical committee guidelines of the ‘Institutional Review Boards/Ethics Committees of Kyushu University Hospital and Medical Institutions’.

## Results

Demographics of the study population

A total of 775 patients were admitted to our department during the study period, with the number of admission attempts being one for 508 individuals and more than one (2 to 14 times) for the remainder (Table 1). We therefore analyzed 1320 hospital admission attempts in total (including failed attempts).

**Table 1:** Number of attempted hospital admissions to the Department of Respiratory Medicine at Kyushu University Hospital during the study period. The number of patients is listed according to the number of attempted hospital admissions during the study period (January 1, 2022, to May 2, 2023).

Number of attempted admissions	Number of patients
1	508
2	140
3	54
4	38
5	15
6	10
7	5
8	2
9	2
14	1
Total number of patients	775
Total number of attempted admissions	1320

Correlation of results for the two PCR targets of Cobas SARS-CoV-2

**Figure 1:**
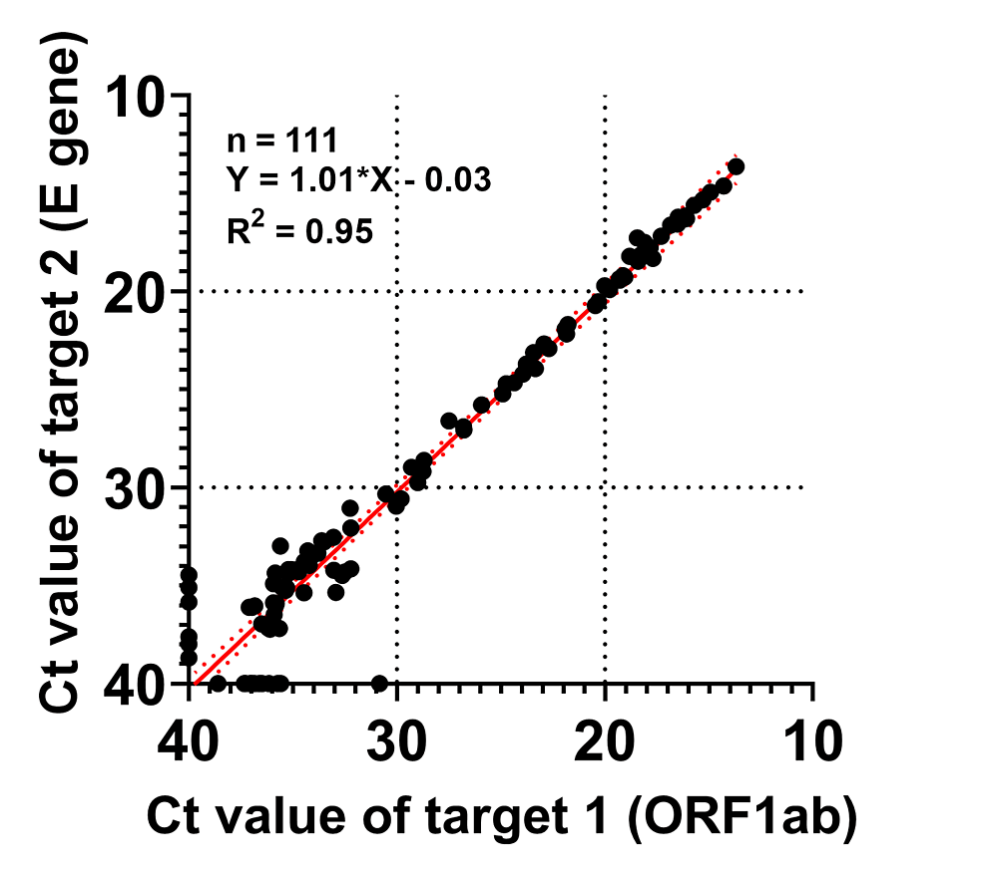
Correlation between the results for the two genomic targets in the Cobas PCR test for SARS-CoV-2. A total of 111 PCR tests showed a positive result for at least one of the two targets, with the Ct value for target 1 (ORF1ab gene) being plotted against that for target 2 (E gene). A negative PCR result was defined as a Ct value of 40. The solid red line shows the linear regression for the 111 PCR results, with the equation of the regression line and R2 value being shown. Six PCR tests were negative for target 1 but positive for target 2, whereas 14 PCR tests were positive for target 1 but negative for target 2.

The Cobas SARS-CoV-2 PCR system targets two genomic regions: the ORF1ab gene (target 1), which is specific for SARS-CoV-2, and the envelope (E) gene (target 2), which is shared by all viruses belonging to the subgenus Sarbecovirus. In general, the results obtained for target 1 and target 2 are well correlated for individuals infected with SARS-CoV-2 [11]. However, target 1 has been found to show low sensitivity for detection of the BA.2.12.1 variant of the virus compared with target 2, presumably as a result of mutations in the ORF1ab gene [12]. We therefore plotted the Ct values of target 1 and target 2 for the 111 Cobas PCR-positive samples (Figure 1). The Ct values of target 1 and target 2 were strongly correlated. Six tests were negative for target 1 but positive for target 2, and 14 tests were positive for target 1 but negative for target 2. Given that the number of negative results for target 1 was less than that for target 2, we decided to further analyze PCR positivity using the results for target 1.

Cobas PCR positivity and false-positive results on admission screening

Among the 1320 attempted hospital admissions, 119 cases were not tested by Cobas PCR. Thirty-nine (3.2%) of the remaining cases yielded a positive PCR result for target 1, including 22 false-positive results and 10 symptomatic COVID-19 cases (Table 2). Seven cases are indeterminate for COVID-19. Thirteen tests (five cases of acute COVID-19 infection, four indeterminate cases, and four false-positive cases) resulted in the cancellation of hospital admission and rescheduling because of the positive PCR results.

**Table 2:** Overall results for Cobas PCR screening of SARS-CoV-2 for hospital admission. A total of 1201 of 1320 cases were screened by Cobas PCR for hospital admission. The screening identified 39 PCR-positive cases and 1162 PCR-negative cases. The 39 PCR-positive cases consisted of 21 false-positive patients who were hospitalized as scheduled, 5 patients with COVID-19 who were hospitalized, 8 false-positive patients whose hospitalization was rescheduled, and 5 patients with COVID-19 whose hospital admission was rescheduled. ^(a)^Frequency was calculated on the basis of the total of 1201 Cobas PCR tests performed. ^(b)^PCR-positive cases indeterminate for COVID-19 were defined in the method.

PCR results of individual hospital admission	No.	%
No PCR test performed or tested by other methods	119	-
PCR-negative at screening	1162	96.8%^(a)^
PCR-positive at screening	39	3.2%^(a)^
False positive with hospital admission as scheduled	18	-
PCR-positive cases indeterminate for COVID-19^(b)^ with hospital admission	3	-
COVID-19 with hospital admission	5	-
False positive with rescheduling of hospital admission	4	-
PCR-positive cases indeterminate for COVID-19^(b)^ with rescheduling of hospital admission	4	-
COVID-19 with rescheduling of hospital admission	5	-
Tested by Cobas PCR	1201	100%^(a)^
Total	1320	-

**Figure 2:**
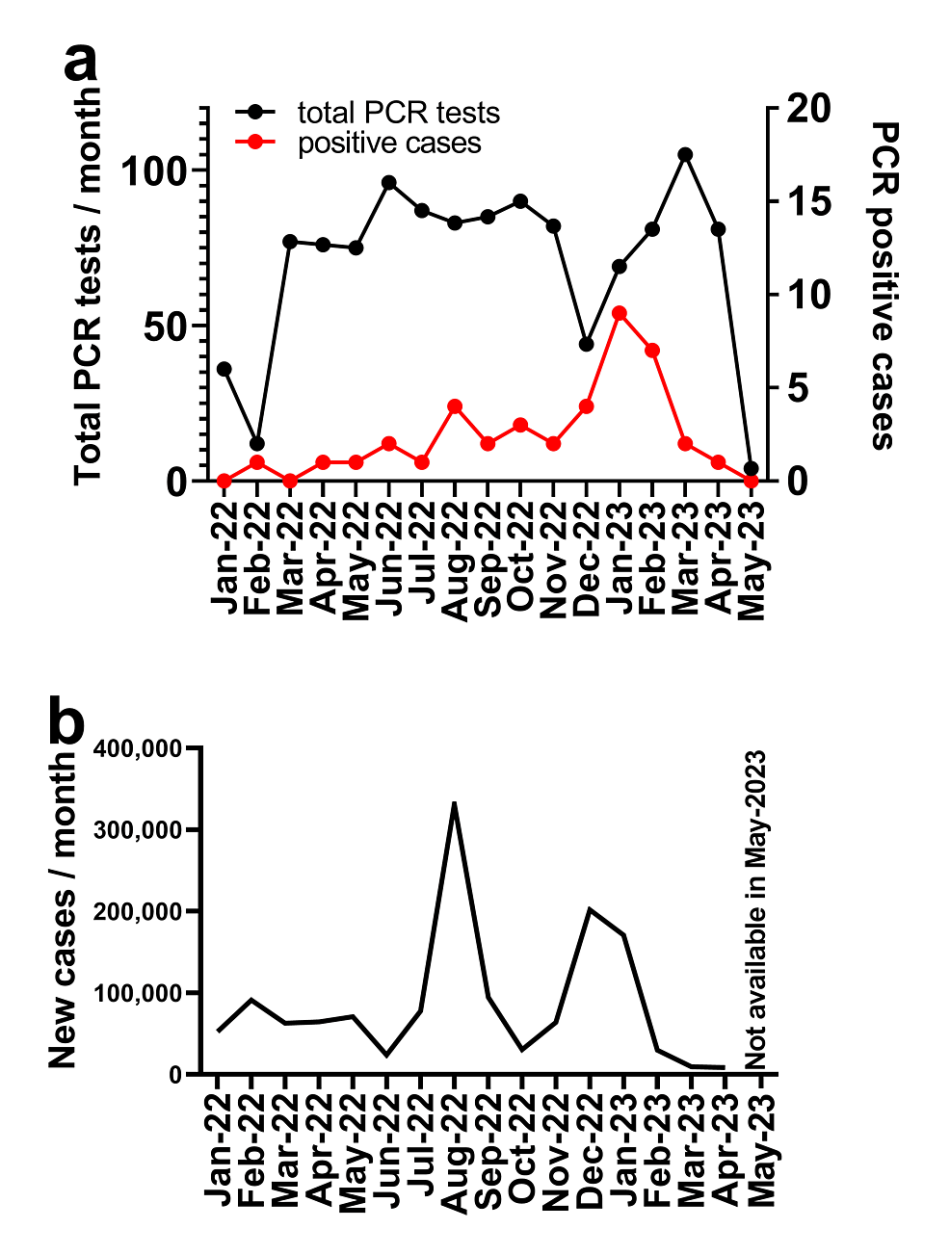
The temporal pattern of the number of PCR screening tests performed for SARS-CoV-2 and the number of positive results obtained during the study period. (a) The black line shows the number of PCR screening tests performed each month during the study period for hospital admission to our department. The red line shows the number of positive PCR results. (b) The monthly COVID-19 cases in Fukuoka prefecture (population of ~5 million) for the same period was calculated from the daily cases reported in a public database (https://www.mhlw.go.jp/stf/covid-19/open-data.html).

The number of PCR-positive cases increased in August 2022 and January 2023 (Figure 2A). The number of PCR screening tests dropped in December 2022 because it was necessary to halt hospital admissions for several weeks as a result of a COVID-19 outbreak in our department. The changes in the number of PCR-positive cases were generally similar to those in the spread of COVID-19 in Fukuoka prefecture (Figure 2B), where our hospital is located. We found that the surge of new COVID-19 cases around December 2022 was one month earlier than our PCR-positive cases, suggesting that PCR tests might detect a recent SARS-CoV-2 infection.

Duration of Cobas PCR positivity after COVID-19

**Figure 3:**
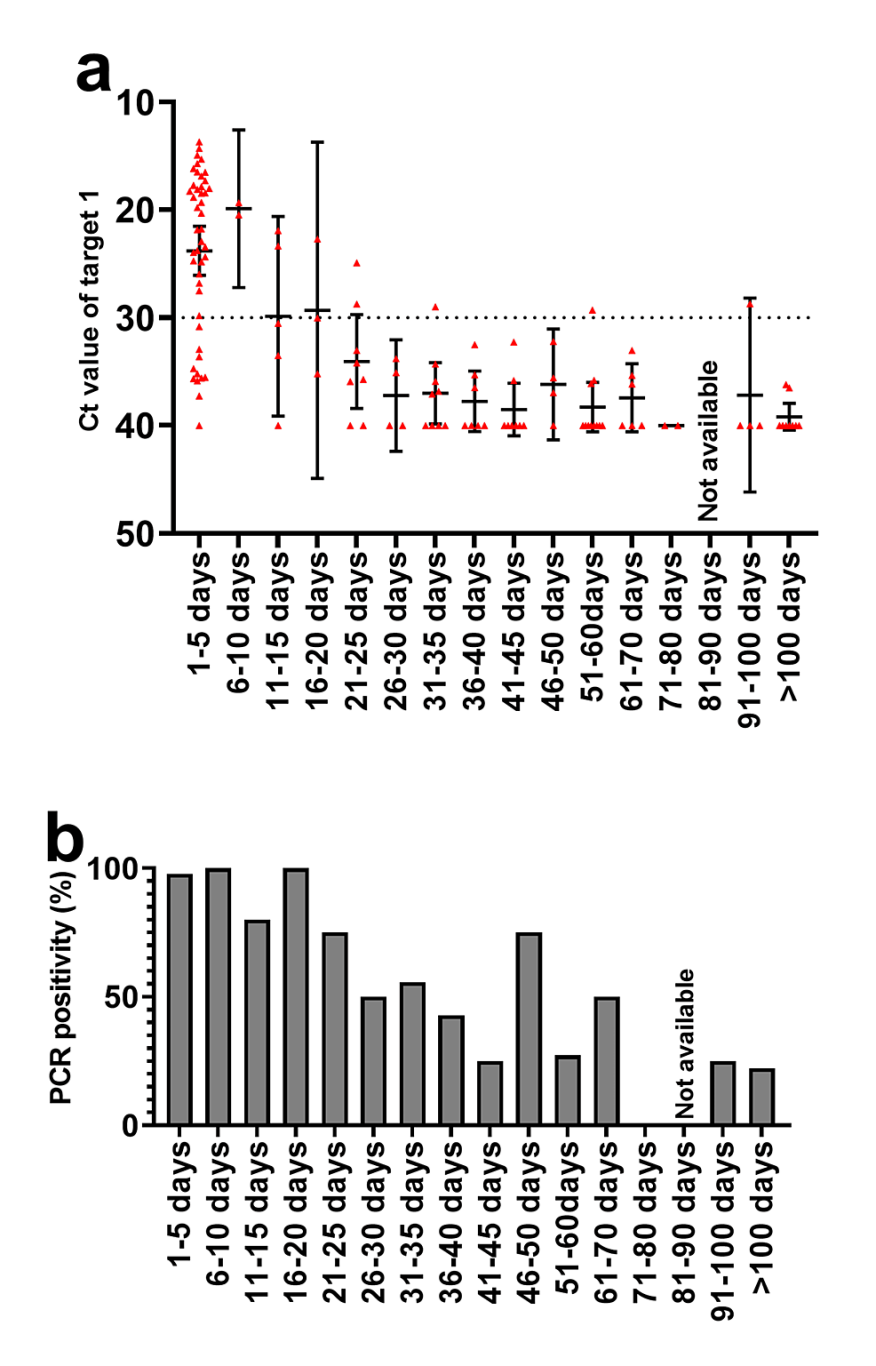
Time course of PCR positivity after COVID-19 onset. (a) Ct values of target 1 for 66 cases of known COVID-19 according to onset date. A negative PCR result was defined as a Ct value of 40. Each red dot corresponds to an individual test result, with the mean and 95% confidence interval being shown. (b) PCR positivity as calculated from the results shown in (a). No PCR test was conducted between 81 and 90 days after COVID-19 onset.

We were able to track Cobas PCR results after the onset of COVID-19 for several patients admitted to our department who were tested more than once. The Ct values of target 1 at one to five days after COVID-19 onset were low (mean ± SD, 23.8 ± 7.6) in general (Figure 3A). Although the mean Ct value of target 1 increased to >30 after day 21 relative to COVID-19 onset, 25% to 75% of Cobas PCR tests remained positive between 21 and 60 days after COVID-19 onset (Figure 3B). To evaluate the relation between patient background and extended PCR positivity, we examined the 23 patients who underwent a Cobas PCR test at 31 to 60 days after COVID-19 onset (Table 3). Nine of the patients did not receive immunosuppressive therapy, whereas the remaining 14 patients received such therapy, including cytotoxic chemotherapy or systemic steroid administration. Among these two groups of patients, one and eight individuals, respectively, showed a positive PCR result between 31 and 60 days after COVID-19 onset.

**Table 3:** Profile of 23 cases tested by PCR at 31 to 60 days after COVID-19 onset. Cases 1 to 9 were not receiving immunosuppressive therapy at the onset of COVID-19. Cases 10 to 23 were receiving immunosuppressive therapy at COVID-19 onset. PCR status is shown as “+” when Cobas PCR testing performed 31 to 60 days after COVID-19 onset was positive at least once; it is shown as “–” when all PCR tests performed between 31 and 60 days after COVID-19 onset were negative.

Case no.	PCR positivity	Comorbidity	Immunosuppressive therapy
1	–	Lung cancer	None
2	–	Lung cancer	None
3	–	Lung cancer	None
4	–	Lung cancer	None
5	–	Lung cancer	None
6	–	Nontuberculous mycobacteriosis	None
7	–	Pneumothorax	None
8	–	Inflammatory lung nodule	None
9	+	Lung cancer	None
10	–	Lung cancer	Dexamethasone
11	–	Lung cancer	Cytotoxic chemotherapy
12	–	Lung cancer	Prednisolone
13	–	Lung cancer	Cytotoxic chemotherapy
14	–	Lung cancer, rheumatoid arthritis	Prednisolone
15	–	Interstitial pneumonia	Prednisolone
16	+	Lung cancer	Cytotoxic chemotherapy
17	+	Lung cancer	Cytotoxic chemotherapy
18	+	Lung cancer	Prednisolone
19	+	Lung cancer	Prednisolone
20	+	Lung cancer	Cytotoxic chemotherapy
21	+	Bronchiolitis obliterans syndrome	Prednisolone
22	+	Interstitial pneumonia	Prednisolone
23	+	Interstitial pneumonia	Prednisolone

## Discussion

We retrospectively evaluated the usefulness and limitations of Cobas PCR screening for SARS-CoV-2 during the process of hospital admission after the emergence of the omicron variant of the virus. A positive PCR result was obtained for 3.2% of cases during such screening, but most of these positive results turned out to be false positives. Nine (43%) of 23 patients tested 31-60 days after COVID-19 onset showed a positive PCR result (Table 3).

Whereas the results obtained for target 1 and target 2 of the Cobas test correlated well overall, there was a slight discrepancy between the two sets of results, especially at high (>30) Ct values (Figure 1). A previous study [12] found low PCR sensitivity for target 1 in the case of the BA.2.12.1 variant, but this variant was not a major circulating strain in Japan at the time of the study [13]. We did not detect a trend toward low sensitivity for target 1.

The number of PCR-positive cases at screening increased slightly in August 2022 and markedly in January 2023, a pattern that matched that for COVID-19 cases in Fukuoka prefecture. Data obtained after September 2023 are not accurate because the Japanese government terminated the requirement to report and track all COVID-19 cases. We experienced 39 Cobas PCR positive results (3.2%) during universal screening for hospital admission. Ten of these cases (0.76% of total tests) were diagnosed as acute-phase COVID-19. Previous studies at other Japanese hospitals have reported 0.07% to 1.81% SARS-CoV-2 positivity for routine screening tests [6-8]. Our PCR positivity rate (0.76%) is thus similar to that of other Japanese studies conducted before the omicron variant outbreak. Even though there were only 10 COVID-19 cases upon hospital admission during the 16 months of screening, we believe that PCR screening was effective in preventing the spread of SARS-CoV-2 into our department. Otherwise, these cases might have been the origin of a hospital outbreak.

Twenty-two of the 39 PCR-positive cases detected during screening for hospital admission were false positives, presumably due to a recent infection. All false-positive cases lacked symptoms then and had a history of COVID-19 in the past four months. From 31 days after COVID-19 onset in our study population, most Ct values were >30, which in general is thought to correspond to a noninfectious state [14]. Some of these cases remained PCR positive at this time. A previous study found that ~15% of patients were PCR-positive 30 days after the onset of COVID-19 [15]. Reinfection may account in part for the relatively high PCR positivity apparent >30 days after initial infection onset. However, reinfection after such a short interval has been found to be rare [16], with previous infections providing protective immunity that can confer up to 90% protection [17,18]. Indeed, we found that patients receiving immunosuppressive therapy were more likely to have a positive PCR result >30 days after COVID-19 onset, presumably as a result of impaired viral clearance. Several studies have suggested that lung cancer [19] or immunosuppressive therapy [20] delays the clearance of the virus. We found some cases remained PCR-positive for up to 100 days (Figures 2A-2B). Additionally, one study [16] reported that the reinfection rate was 4.2%, occurring 122 to 674 days after the initial infection (mean of 507 days). Given these findings and previous studies, we defined asymptomatic PCR-positive cases with a COVID-19 history within the past four months as false positives. We experienced asymptomatic PCR-positive cases lacking recent COVID-19 history in the last four months. We defined these cases as ‘PCR positive cases indeterminate for COVID-19’ because we could not figure out the reason for PCR positivity. We assume that these cases are possibly asymptomatic infections, but we cannot prove this due to the nature of the retrospective observational study. Universal PCR screening is effective for the identification and isolation of individuals with COVID-19 on hospital admission. However, hospital physicians need to be aware of the possibility of false-positive results that are due to previous infections, especially if SARS-CoV-2 infection in the local population was prominent several weeks ago. The combination of a history of recent infection and a high (>30) Ct value can help to distinguish a false-positive PCR result from acute-phase COVID-19. Most PCR screening studies upon hospital admission were conducted before the omicron strain outbreak, and there was little chance to come across false-positive cases due to recent COVID-19. We believe our study is informative to discuss the policy of COVID-19 screening in the respiratory medicine department.

Limitations

Our research has several limitations. First, some COVID-19 cases were diagnosed using alternative PCR methods, particularly during emergency hospital admissions at night when the Cobas PCR was unavailable. This may affect the total number of COVID-19 cases reported. Second, our study was a retrospective observational study. We cannot determine the reasons for indeterminate COVID-19 cases because our information was insufficient to distinguish between asymptomatic COVID-19 and previously undocumented COVID-19. Third, our study was conducted in a single department of a university hospital, which mainly accepts lung cancer patients. These patients were highly sensitive to COVID-19 infection and often visited other clinics for PCR or antigen tests, which might affect the proportion of false positives and confirmed COVID-19 cases upon our hospital admission. Fourth, the sample size was smaller than in the previous study (over 5,000 tests) because the PCR screening policy was conducted only in our department.

## Conclusions

PCR screening tests for SARS-CoV-2 upon hospital admission are an effective measure to identify and avoid SARS-CoV-2 flow into the hospital. However, an immunocompromised host may show a false positive PCR test from 31 to 60 days post-infection. We need to judge the PCR test result based on the recent COVID-19 history and Ct value.

## Appendices

Raw data in this study is deposited at figshare under the address of “https://figshare.com/articles/dataset/Utility_and_limitation_of_universal_PCR_screening_for_SARS-CoV-2_on_hospital_admission/24416323.”
